# Association of QT interval-prolonging drugs with clinical trial eligibility in patients with advanced cancer

**DOI:** 10.3389/fcvm.2022.894623

**Published:** 2022-12-15

**Authors:** Elizabeth J. Rowe, Tyler Shugg, Reynold C. Ly, Santosh Philips, Marc B. Rosenman, John T. Callaghan, Milan Radovich, Brian R. Overholser, Bryan P. Schneider, James E. Tisdale, Todd C. Skaar

**Affiliations:** ^1^Division of Clinical Pharmacology, Department of Medicine, Indiana University School of Medicine, Indianapolis, IN, United States; ^2^Ann & Robert H. Lurie Children’s Hospital of Chicago, Feinberg School of Medicine, Northwestern University, Chicago, IL, United States; ^3^Department of Pharmacology and Toxicology, Indiana University School of Medicine, Indianapolis, IN, United States; ^4^Division of Hematology/Oncology, Department of Medicine, Indiana University School of Medicine, Indianapolis, IN, United States; ^5^Department of Pharmacy Practice, Purdue University College of Pharmacy, West Lafayette, IN, United States

**Keywords:** cancer, clinical trial eligibility, clinical trial exclusion, QT interval, QT-prolonging drugs, QTc

## Abstract

**Introduction:**

Drug-induced prolongation of the heart rate-corrected QT interval (QTc) is associated with increased risk for the potentially fatal arrhythmia torsades de pointes. Due to arrhythmia risk, clinical trials with cancer therapeutics often exclude patients based on thresholds for QTc prolongation. Our objective was to assess associations between prescriptions for QT-prolonging drugs and the odds of meeting cancer trial exclusionary QTc thresholds in a cohort of adults with advanced cancer.

**Methods:**

Electronic health records were retrospectively reviewed for 271 patients seen at our institutional molecular solid tumor clinic. Collected data included demographics, QTc measurements, ventricular arrhythmia-related diagnoses, and all inpatient and outpatient prescriptions. Potential associations were assessed between demographic and clinical variables, including prescriptions for QT-prolonging drugs, and QTc measurements.

**Results:**

Women had longer median QTc measurements than men (*p* = 0.030) and were prescribed more QT-prolonging drugs during the study (*p* = 0.010). In all patients, prescriptions for QT-prolonging drugs were associated with longer median and maximum QTc measurements at multiple assessed time points (i.e., for QT-prolonging drugs prescribed within 10, 30, 60, and 90 days of QTc measurements). Similarly, the number of QT-prolonging drugs prescribed was correlated with longer median and maximum QTc measurements at multiple time points. Common QTc-related exclusionary criteria were collected from a review of ClinicalTrials.gov for recent cancer clinical trials. Based on common exclusion criteria, prescriptions for QT-prolonging drugs increased the odds of trial exclusion.

**Conclusion:**

This study demonstrates that prescriptions for QT-prolonging drugs were associated with longer QTc measurements and increased odds of being excluded from cancer clinical trials.

## Introduction

Drug-induced prolongation of the QT interval on the surface electrocardiogram (ECG), which corresponds to the period in which cardiac ventricular depolarization and repolarization occur, is associated with an increased risk of potentially fatal ventricular arrhythmias, including torsades de pointes (TdP) (1). QT interval length reflects a balance between depolarizing and repolarizing ionic currents in the ventricle, and drugs that prolong the QT interval do so by affecting the function of ventricular currents, most commonly *via* inhibition of the rapid component of the delayed rectifier potassium current (I_Kr_) (2). The heart rate-corrected QT interval (QTc) is an established monitoring parameter to assess the risk of drug-induced TdP both in the clinical setting (3) and during development and regulatory approval of new medications (4).

The QTc interval is also frequently used as a criterion for clinical trial eligibility, including in cancer, where a number of efficacious treatment options have been demonstrated to prolong QTc (5). A multitude of ongoing cancer trials in the United States (US) have exclusion criteria based on QTc thresholds (as listed on ClinicalTrials.gov), potentially preventing cancer patients from receiving life-saving therapies. While exclusion of patients at increased risk of potentially fatal arrhythmias may be warranted, clinical guidance is available to manage drug-induced arrhythmia risk (3, 6, 7), including specific recommendations for cancer patients (5, 8). One common strategy to reduce the risk of drug-induced arrhythmias is discontinuation of concomitant medications that prolong QTc (7, 8). For non-antiarrhythmics, alternative therapies often exist, even within the same medication class, that do not prolong QTc (9). Therefore, therapeutic substitution to reduce the number of QT-prolonging drugs may be a viable strategy to prevent exclusion of patients from clinical trials, particularly since past investigations have found that concomitant administration of multiple QT-prolonging drugs produced incremental increases in QTc prolongation (10, 11).

The potential for the administration of QT-prolonging drugs to affect clinical trial eligibility is supported by numerous investigations that have demonstrated high rates of prescriptions for QT-prolonging drugs in cancer patients (12–15). Moreover, various cancer therapies, including many tyrosine kinase inhibitors (TKIs), result in clinically relevant QTc prolongation (16–19). However, the impact of QT-prolonging drugs on trial eligibility has not been directly studied. Accordingly, the purpose of this research was to assess the potential for drug-induced QTc prolongation to affect clinical trial eligibility within a cohort of adult patients with advanced cancer. Our specific objectives included the following: (1) to survey study protocols for ongoing or recently completed cancer clinical trials, in order to document their exclusionary QTc thresholds; (2) to determine associations between demographic factors and administration of QT-prolonging drugs with QTc values obtained from electronic health records (EHRs); and (3) to assess the impact of demographic factors and administration of QT-prolonging drugs on clinical trial eligibility based on the exclusionary QTc thresholds used by cancer clinical trials and recommended by professional organizations.

## Materials and methods

### Patient enrollment and eligibility

Our study population consisted of adult patients with advanced solid cancers who were treated at the Indiana University Health Precision Genomics Clinic in Indianapolis, Indiana, US and enrolled in the Indiana University Total Cancer Care Protocol (part of the larger Oncology Research Information Exchange Network-wide Total Cancer Care initiative). Patients enrolled in the Total Cancer Care Protocol were selected for inclusion in this study if their EHR included, after their date of diagnosis of cancer, at least one Bazett’s-corrected QT value and administration of at least one medication. Bazett’s correction was used throughout our analyses since, relative to other correction methods, it has the strongest data associating QTc threshold values with arrhythmia risk (3). The EHR data were obtained *via* query of the Indiana Health Information Exchange, a state-wide EHR repository with data from 38 health systems. Using these criteria, we identified 275 eligible patients. We excluded four patients since their only QTc measurements were those taken within 1 day of death or cardiac resuscitation (2 patients) or after they had been implanted with implantable cardioverter-defibrillators (ICDs) with functioning ventricular pacemakers (2 patients). As a result, our final cohort included 271 patients who were enrolled at clinic visits between February 2015 and February 2018 (Supplementary Figure 1 in Data Sheet 2). The research protocols for this study and the parent Total Cancer Care Protocol were approved by the Indiana University Institutional Review Board, and all patients provided written informed consent.

### Survey of corrected QT eligibility requirements in clinical trials

Using the ClinicalTrials website^1^ (20), which is maintained by the US National Library of Medicine, we conducted a survey of clinical trial eligibility requirements related to exclusionary QTc thresholds. We searched for oncology trials involving any pharmacotherapeutic intervention, as well as specifically for trials including the following TKIs that are known to prolong the QT interval: bosutinib, cabozantinib, ceritinib. cobimetinib, crizotinib, dabrafenib, dasatinib, encorafenib, entrectinib, gilteritinib, lapatinib, lenvatinib, necitumumab, nilotinib, osimertinib, pazopanib, sorafenib, sunitinib, vandetanib, or vemurafenib. We limited our search to trials available within the US that were enrolling patients between January 1, 2010 and December 31, 2020 to match our study population. We further limited our search to protocols that contained the keyword “QT.” We then manually reviewed each protocol to identify QTc values that served as exclusionary thresholds.

### Study data collection and classification

Electronic health record (EHR) data were obtained from the Indiana Health Information Exchange through April 20, 2020 and included demographic data (age, date of first cancer diagnosis, date of death, sex, and race), all inpatient and outpatient prescriptions, QTc measurements, and ventricular arrhythmia-related diagnoses and interventions (list of queried International Classification of Diseases and Current Procedural Terminology codes provided in Supplementary Table 1 in Data Sheet 2). All prescriptions, QTc measurements, diagnoses, and interventions had associated dates. In addition, prescription data included the dispensing location (i.e., whether administered in a medical setting, including outpatient clinics, or whether dispensed from an outpatient pharmacy). Within our analyses, we classified medications as “QT-prolonging” if they were categorized by the FDA-supported CredibleMeds^®^ database^2^ as having a “known” or “possible” risk of TdP (9). All other medications were classified for our purposes as “non-QT-prolonging.” Medications classified by CredibleMeds^®^ as having a “conditional risk of TdP,” meaning that they do not independently prolong QT but can trigger clinical conditions that lead to QT prolongation (e.g., thiazide diuretic-induced hypokalemia), were not considered as “QT-prolonging” in our analyses; this decision was made since evidence of the associated QT-prolonging conditions was not routinely collected in the EHR, which did not allow us to verify whether the conditions were met for these drugs to prolong QT.

QTc measurements were collected for each patient since their respective date of first cancer diagnosis. We then reviewed the dates of QTc measurements relative to interventions or diagnoses that may be associated with alterations to QTc. QTc measurements that occurred (1) within 24 h of cardiac arrest or death from any cause or (2) any time after placement of cardioverter-defibrillator or pacemaker devices were excluded. From the remaining values for each individual, we calculated the maximum, minimum, median, and mean QTc measurements, and the difference between maximum and minimum QTc measurements, termed the delta QTc. We determined whether each individual’s QTc measurements exceeded QTc thresholds from our survey of ClinicalTrials.gov or those established as potentially proarrhythmic by scientific statements from the American Heart Association (AHA) and the American College of Cardiology (ACC): 450 ms for men and 460 ms for women (the 95th percentile of normal QTc variation); 470 ms for men and 480 ms for women (the 99th percentile); and 500 ms in both sexes (3, 21).

### Association of QT-prolonging drugs with QTc values

For each patient, the date of maximum QTc was considered the index date. We then determined how many drugs were prescribed within 10, 30, 60, or 90 days before the index date. We categorized the patients by whether they had been prescribed QT-prolonging drugs within each time period before the index date, or only non-QT prolonging drugs (or no drugs at all).

Our prescription data did not include the days’ supply. Therefore, within our paired analysis that compared QTc values in patients while taking and not taking QT-prolonging drugs, the following assumptions were used to conservatively determine the day’s supply. For prescriptions administered in a medical setting, the days’ supply was assumed to be one. For prescriptions dispensed from an outpatient pharmacy, the days’ supply was assumed based on the shortest days’ supply for indications for which the drug is commonly prescribed (see Supplementary Table 2 in Data Sheet 2 for a complete list of assumed durations for all prescriptions dispensed from a pharmacy). An exception to this method was made for prescriptions dispensed from a pharmacy that were (1) dispensed for at least three consecutive regular intervals (e.g., every 30 days, every 90 days) and (2) written for medications that are commonly used as maintenance therapy for chronic medical conditions (e.g., antihypertensives). For these prescriptions, the patient was assumed to be taking the medication for the entire interval between consecutive prescriptions.

### Statistical analysis

We used the Kruskal-Wallis test by ranks to investigate differences in continuous QTc-related variables (maximum, minimum, mean, median, and delta QTc) between patients grouped by discrete independent variables (e.g., patient race, whether patients were prescribed QT-prolonging drugs). When more than two discrete independent variables were compared, we performed a *post hoc* Dunn’s Test with Bonferroni correction to determine which groups were different from each other. We used Spearman’s rank correlations to evaluate correlations between continuous independent and dependent variables (e.g., patient age and maximum QTc). We used Chi-squared tests to investigate correlations between discrete independent and dependent variables (e.g., patient sex and whether median QTc values met various QTc thresholds). Finally, we used logistic regression to evaluate correlations with binary dependent variables, such as determining how the number of QT-prolonging drugs affected the odds of meeting exclusionary QTc thresholds. Results were considered significant at *p* < 0.05 or less than adjusted *p*-value thresholds after Bonferroni correction from *p* = 0.05.

## Results

### Survey of QTc eligibility requirements from ClinicalTrials.gov

**FIGURE 1:**
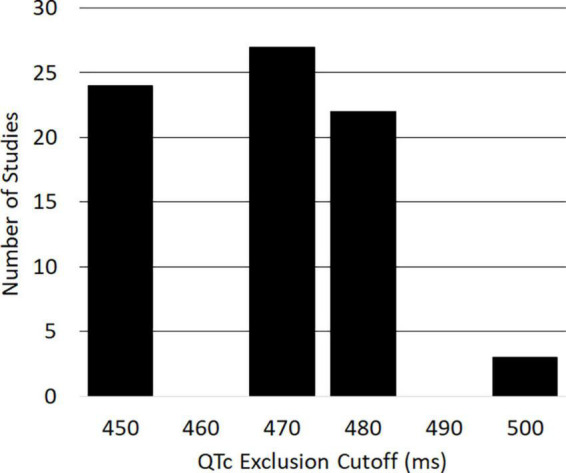
Histogram of corrected QT (QTc) measurements used as exclusion criteria in United States clinical oncology trials from 2010 to 2020 (*n* = 76).

Limiting our search of the ClinicalTrials.gov database to trials conducted in the US between 2010 and 2020, we found 158 clinical trials for oncology therapeutics that specifically mentioned QT prolongation in their protocols. Of these, *n* = 93 included the QT interval in their eligibility criteria; the remaining studies instead mentioned QT as an outcome measure. Of the 93 studies that used QT for inclusion or exclusion criteria, 37 studies excluded participants with a family or personal history of congenital long QT syndrome, and 34 studies prohibited patients from taking QT-prolonging drugs while on study. Seventy-six of the studies provided specific QTc thresholds that potential trial patients could not exceed in order to be eligible (distribution of thresholds shown in Figure 1). Five of these studies (6.6%) included sex-specific QTc thresholds, which consisted of 450 ms for men and 470 ms for women. These studies are represented using their least stringent QTc threshold (470 ms) in Figure 1. The identified exclusionary QTc thresholds were 450 ms (31.6% of studies), 470 ms (35.5%), 480 ms (28.9%), and 500 ms (3.9%), which correspond to clinically relevant QTc values established by AHA/ACC scientific statements.

### Summary of patient demographic and clinical data

Our cohort consisted of 271 adults with advanced cancer who had at least one medication prescription and QTc measurement since their respective date of first cancer diagnosis. As displayed in Table 1, our cohort was 58 (49, 64) [median (1st quartile, 3rd quartile)] years old, was evenly split by sex (50.9% female), and was mostly white (88.9%). The most common cancer types at first diagnosis were pancreatic (12.9%), breast (9.6%), and colorectal (9.2%). Ventricular arrhythmia-related diagnoses occurred in 9 patients (3.3%) and included ventricular tachycardia and cardiac arrest, which occurred in 6 and 3 patients, respectively. The rate of ventricular arrhythmias was higher in our cohort than those estimated in the general population for similarly aged individuals (18, 22). This may be attributable to the facts that many cancer therapies can cause ventricular arrhythmias (23) or that advanced cancer patients have an increased risk of ventricular arrhythmias relative to those with less advanced disease (24). Serum electrolyte abnormalities known to prolong the QT interval were common in our cohort, with the incidence of at least one episode of hypocalcemia, hypokalemia, and hypomagnesemia being 86.3, 83.0, and 68.3%, respectively. Of the 28 subjects with index QTc measurements > 500 ms and serum electrolyte concentrations from that same day, 18 (64.3%) had a serum electrolyte abnormality that may have contributed to their prolonged index QTc. The median heart rate was 83 (72, 96) beats per minute, and 92.6 and 85.6% of the cohort experienced at least one episode of tachycardia and bradycardia, respectively. Three patients (1.1%) had a medical history that included placement of an ICD or pacemaker. Fifty-four patients (19.9%) had recorded dates of death during the study period. The median duration of follow-up, defined as the elapsed time between the date of first cancer diagnosis and the date of any last study event (e.g., prescription, QT measurement), was 3.0 (1.2, 5.8) years. Since first cancer diagnosis, our cohort had a total of 19,306 unique prescriptions for QT-prolonging drugs with a median of 8 [6, 10] unique drugs per patient. Of the 271 patients in our cohort, 270 (99.6%) had ≥ 1 prescription for a QT-prolonging drug since first cancer diagnosis. In addition, our cohort had a total of 1,164 unique QTc measurements since first cancer diagnosis, with a median of 3 (2, 6) QTc measurements per patient. The median QTc for our cohort was 438 (423, 453) ms, and the minimum and maximum QTc values were 320 and 633 ms, respectively; the median index QTc, defined as the maximum QTc at the patient level, was 456 (437.5, 478) ms.

**TABLE 1:** Demographic and clinical characteristics of patients with advanced cancer included in the study.

Variable	Value in study cohort (*n* = 271)
Age in years at first cancer diagnosis [median (quartile 1, quartile 3)]	58 (49, 64)
Number of patients age ≥ 65 years [count (percent)]	65 (24.0%)
Duration of follow-up in years* [median (quartile 1, quartile 3)]	3.0 (1.2, 5.8)
*Sex [count (percent)]*	
Female	138 (50.9%)
Male	133 (49.1%)
*Race [count (percent)]*	
White	241 (88.9%)
Black	26 (9.6%)
Asian	4 (1.5%)
*Cancer type at first diagnosis [count (percent)]*	
Pancreatic	35 (12.9%)
Breast	26 (9.6%)
Colorectal	25 (9.2%)
Soft-tissue sarcoma	24 (8.9%)
Prostate	23 (8.5%)
Ovarian	13 (4.8%)
Renal	10 (3.7%)
Non-small cell lung	9 (3.3%)
Cholangiocarcinoma	8 (3.0%)
Head and neck	8 (3.0%)
Unknown primary	8 (3.0%)
*Ventricular arrhythmia-related diagnoses [count (percent)]*	
Ventricular tachycardia	6 (2.2%)
Cardiac arrest	3 (1.1%)
*Serum electrolyte abnormalities^+^ [count (percent)]*	
Hypocalcemia (< 8.5 mg/dL, ionized < 4.5 mg/dL)	234 (86.3%)
Hypokalemia (< 3.5 mEq/L)	225 (83.0%)
Hypomagnesemia (< 1.7 mg/dL)	185 (68.3%)
*Heart rate values [in bpm or count(percent)]*	
Heart rate [median (quartile 1, quartile 3)]	83 (72, 96)
Bradycardia (< 60 bpm)	232 (85.6%)
Tachycardia (> 100 bpm)	251 (92.6%)
Placement of implantable cardiac defibrillator or pacemaker [count (percent)]	3 (1.1%)
All-cause mortality during study [count (percent)]	54 (19.9%)
*Corrected QT (QTc) values (in ms)*	
Median QTc [median (quartile 1, quartile 3)]	438 (423, 453)
Minimum QTc	320
Maximum QTc	633
Index QTc^++^ [Median (Quartile 1, Quartile 3)]	456 (437.5, 478)

*Duration of follow-up was defined as the time elapsed between the date of first cancer diagnosis and date of most recent prescription.

^+^The incidence of serum electrolyte abnormalities was assessed based on diagnoses and on lab values below the specified thresholds.

^++^Index QTc was defined as the maximum observed QTc for each individual patient.

### Association of patient demographics with prescriptions for QT-prolonging drugs and QTc values

Women were prescribed more QT-prolonging medications than men when the overall study period was considered [median: 8 QT-prolonging drugs, 1st and 3rd quartiles: (6, 10) for women versus 7 (6, 9) for men, *p* = 0.010]. Similarly, patients younger than age 65 were prescribed more QT-prolonging drugs during the overall study period than those over 65 [8 (6, 10) versus 7 (5, 9), *p* = 0.006], and age was inversely correlated with the number of QT-prolonging drugs prescribed (Spearman’s ρ = −0.26, *p* < 0.001). However, when the timing of prescriptions relative to the index date was considered, women and those under 65 were not more likely to be prescribed a QT-prolonging drug within 90, 60, 30, or 10 days before the index date. Demographic characteristics were not otherwise associated with the number of QT-prolonging drugs prescribed, nor were cardiac arrest, ventricular arrhythmia-related diagnoses, ICD/pacemaker implant, or patient death during the study period.

Women had significantly longer median QTc measurements than men [442 (426, 456) ms versus 435 (422, 448) ms, *p* = 0.030]. Men and women did not differ with regard to other measures of QTc (i.e., mean QTc, maximum QTc, minimum QTc, and delta QTc), and significant differences in QTc were not observed when patients were grouped by other demographic characteristics (i.e., age at first cancer diagnosis, age greater than 65, race, or cancer diagnosis). Patients who experienced cardiac arrest or who died during the study period did not differ with regard to their QTc measurements. Patients with ICDs or pacemakers (*n* = 3) had longer delta QTcs (*p* = 0.010), but did not differ with regard to other measures of QTc. Patients with ventricular arrhythmias (*n* = 9) had longer maximum QTc measurements than patients who did not [517 (495, 518) ms versus 456 (436, 476) ms, *p* < 0.001] but did not differ with regard to other QTc measures.

### Association of QT-prolonging drugs with QTc values

**FIGURE 2:**
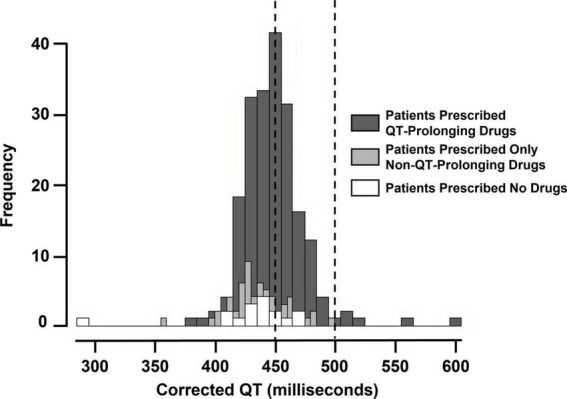
Histogram of maximum corrected QT (QTc) measurements (i.e., index QTc) in patients prescribed QT-prolonging drugs, only non-QT-prolonging drugs, and no drugs within 30 days before the index date. The vertical dashed lines at 450 and 500 milliseconds indicate minimum and maximum cancer clinical trial exclusionary QTc thresholds.

We assessed whether prescriptions for QT-prolonging drugs were associated with QTc values. A histogram of QTc measurements stratified by whether patients were prescribed QT-prolonging drugs within 30 days of the index date is shown in Figure 2, with quantitative results provided in Table 2. Results for the other analyzed time points (90 days, 60 days, 10 days, any time before) are shown in Supplementary Table 3 in Data Sheet 2. Median QTc measurements were significantly longer in patients prescribed QT-prolonging drugs [442 (425, 456) ms] within 30 days of the index date relative to those prescribed only non-QT prolonging drugs [427 (418, 440) ms] or no drugs at all [432 (423, 445) ms; *p* < 0.001]. Similar associations were also observed for maximum QTc (*p* < 0.001) and delta QTc (*p* = 0.002), and we observed similar patterns of higher QTc measurements in patients prescribed QT-prolonging medications within 10, 60, 90 days, or any time before their index date. Further, the number of QT-prolonging drugs prescribed within 30 days of the index date was correlated with median QTc (Spearman’s ρ = 0.20, *p* = 0.001; Table 3) as well as maximum QTc (*p* < 0.001) and delta QTc (*p* < 0.001); these associations were also observed at the other assessed time points. Minimum QTc was not associated with prescriptions for QT-prolonging drugs and was not correlated with the number of QT-prolonging drugs prescribed at any time point. When considering individual drugs prescribed within 30 days of the index date, patients with prescriptions for ondansetron (*p* = 0.005), promethazine (*p* = 0.013), or propofol (*p* = 0.043) had higher median QTc measurements than patients not prescribed each of these drugs (Table 4); these were also the three most commonly prescribed QTc prolonging drugs in our cohort.

**TABLE 2:** Corrected QT (QTc) measures based on whether patients were prescribed QT-prolonging drugs within 30 days of the index date.

	Patients prescribed no drugs (*n* = 15)	Patients prescribed only non-QT drugs (*n* = 57)	Patients prescribed QT-prolonging drugs (*n* = 199)	*P*-value (*post hoc P*-value for QT vs. non-QT)
Mean QTc	438 (423, 443)	429 (418, 443)	443 (429, 457)	**< 0.001 (< 0.001)**
Median QTc	432 (423, 445)	427 (418, 440)	442 (425, 456)	**< 0.001 (0.001)**
Maximum QTc	441 (426, 477)	437 (421, 469)	460 (445, 482)	**< 0.001 (< 0.001)**
Minimum QTc	424 (420, 440)	416 (405, 430)	421 (406, 442)	0.080 (0.080)
Difference between maximum and minimum QTc (Delta QTc)	0 (0, 24)	17 (0, 37)	37 (11, 64)	**< 0.001 (0.002)**

All values are in milliseconds. All data are presented as: median (1st quartile, 3rd quartile). Kruskal-Wallis test was used to compare continuous data. Bold values indicates that the *p*-value is significant at the <0.05 threshold.

**TABLE 3:** Correlation between the number of prescribed QT-prolonging drugs and corrected QT (QTc) measures at assessed time points.

	Any time before	90 days	60 days	30 days	10 days
**Spearman correlation: ρ (*p*-value)**					
Mean QTc	**0.15 (0.020)**	**0.21 (< 0.001)**	**0.22 (< 0.001)**	**0.20 (0.001)**	**0.19 (0.002)**
Median QTc	**0.14 (0.020)**	**0.19 (0.002)**	**0.21 (< 0.001)**	**0.20 (0.001)**	**0.19 (0.002)**
Maximum QTc	**0.24 (< 0.001)**	**0.31 (< 0.001)**	**0.32 (< 0.001)**	**0.28 (< 0.001)**	**0.28 (< 0.001)**
Minimum QTc	−0.01 (0.89)	0.00 (0.94)	0.02 (0.70)	0.02 (0.70)	−0.01 (0.92)
Difference between maximum and minimum QTc (Delta QTc)	**0.29 (< 0.001)**	**0.38 (< 0.001)**	**0.36 (< 0.001)**	**0.26 (< 0.001)**	**0.31 (< 0.001)**

Bold values indicates that the *p*-value is significant at the <0.05 threshold.

**TABLE 4:** Most commonly prescribed QT-prolonging drugs, ranked by number of patients prescribed the drug at 30 days before the maximum corrected QT (QTc) index date, with associated median QTc values and number of patients that exceeded QTc thresholds.

	All patients				Patients meeting maximum QTc > 450/450 ms threshold		Patients meeting maximum QTc > 470/480 ms threshold		Patients meeting maximum QTc > 500 ms threshold	
				
Drug name	Number of Patients (%)	Median QTc for patients on drug	Median QTc for patients not on drug	*P*-value	Patients on drug	*P*-value	Patients on drug	*P*-value	Patients on drug	*P*-value
Ondansetron	159 (58.7%)	443 ± 31	433 ± 29	**0.005**	97 (61.0%)	**< 0.001**	58 (36.5%)	**< 0.001**	26 (9.6%)	**0.003**
Promethazine	101 (37.3%)	446 ± 32	435 ± 28	**0.013**	61 (60.4%)	**0.024**	33 (32.7%)	0.16	13 (4.8%)	0.56
Propofol	34 (12.5%)	449 ± 40	437 ± 27	**0.043**	25 (73.5%)	**0.006**	14 (41.2%)	0.067	5 (1.8%)	0.56
Palonosetron	29 (10.7%)	446 ± 20	438 ± 31	0.39	14 (48.3%)	0.85	5 (17.2%)	0.27	3 (1.1%)	1.00
Azithromycin	20 (7.4%)	446 ± 41	438 ± 29	0.41	15 (75.0%)	**0.036**	10 (50%)	**0.034**	4 (1.5%)	0.26
Tramadol	17 (6.3%)	438 ± 35	438 ± 30	0.94	9 (52.9%)	1.00	4 (23.5%)	0.79	2 (0.7%)	1.00
Ciprofloxacin	17 (6.3%)	440 ± 29	438 ± 30	0.54	10 (58.8%)	0.62	6 (35.3%)	0.58	3 (1.1%)	0.42
Levofloxacin	12 (4.4%)	443 ± 14	438 ± 31	0.41	10 (83.3%)	**0.035**	7 (58.3%)	**0.022**	5 (1.8%)	**0.006**
Escitalopram	10 (3.7%)	438 ± 23	438 ± 31	0.78	4 (40%)	0.53	3 (30%)	1.00	0 (0%)	1.00
Mirtazapine	8 (3%)	442 ± 15	438 ± 31	0.90	4 (50%)	1.00	2 (25%)	1.00	1 (0.4%)	1.00

All QTc values are in milliseconds. Data are presented as median ± interquartile range and percentages. Kruskal-Wallis test was used to compare continuous data, and Fisher’s exact test was used to determine if the proportion of patients who exceeded a given QTc exclusionary threshold was higher among patients prescribed the drug compared to patients not prescribed the drug. AHA/ACC scientific statements identify 450 ms (men)/460 ms (women) and 470 ms (men)/480 ms (women) as the 90th and 99th percentiles of the normal QTc intervals, respectively. QTc > 500 ms for all patients was identified as a relevant QTc threshold by both the AHA/ACC and from our survey of ClinicalTrials.gov. Due to space issues, only the QTc thresholds identified from the AHA/ACC are shown on this table. Associations between QT-prolonging drugs and the proportions of patients exceeding QTc thresholds from our survey of ClinicalTrials.gov is shown in Supplementary file 1. Bold values indicates that the *p*-value is significant at the <0.05 threshold.

**FIGURE 3:**
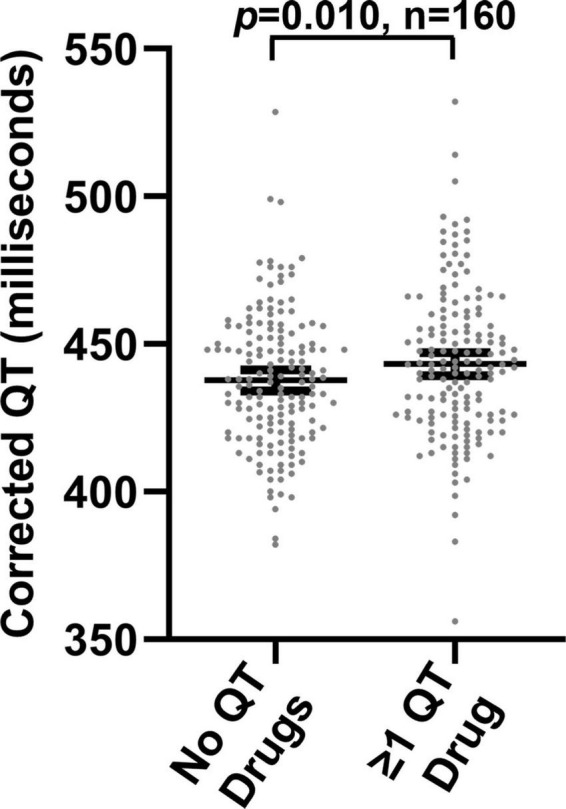
Median corrected QT measurements in 160 patients in the absence and presence of concomitant prescriptions for one or more QT-prolonging drugs.

We also performed a paired analysis in 160 patients who had QTc measurements both during and not during concomitant treatment with ≥ 1 QT-prolonging drug. For this analysis, we assigned each medication prescription a days’ supply based on the type of medication and the observed prescribing patterns (see methods for additional details), and we assessed the days’ supplies for temporal overlap with QTc measurements. As illustrated in Figure 3, median QTc values were longer in patients when concomitantly prescribed ≥ 1 QT-prolonging drug (mean of medians: 443.2 ms) than when not co-prescribed QT-prolonging drugs (mean of medians: 437.7; *p* = 0.010). A histogram displaying changes in median QTc measurements for each individual patient during concomitant treatment with QT-prolonging drugs (relative to when not treated with QT-prolonging drugs) is shown in Supplementary Figure 2 in Data Sheet 2.

### Association of patient demographics and prescriptions for QT-prolonging drugs with clinical trial exclusion

Based on our findings from surveying the ClinicalTrials.gov database, the number of patients in our cohort meeting common clinical trial exclusionary QTc thresholds is shown in Table 5. Overall, 27.3 and 57.9% of our cohort would be excluded from clinical trials based on their median and maximum QTc values, respectively, when applying the most stringent exclusionary QTc threshold (450 ms). In addition, 11.4% of our cohort had maximum QTc values that exceeded 500 ms, which corresponds to the least stringent exclusionary QTc threshold and is described in AHA/ACC scientific statements as being “dangerously” proarrhythmic. Women in our study were more likely to have median QTc values that exceeded the 450 ms threshold than men (33.3% of women versus 21.1% men, *p* = 0.030). This is notable given that < 10% of surveyed cancer trials had sex-specific exclusionary QTc thresholds and 31.6% of trials used a 450 ms threshold for all patients. If these trials used sex-specific thresholds at the 95th percentile described in the AHA/ACC scientific statements (i.e., 450 ms for men and 460 ms for women), only 15.9% of women in our cohort (rather than 33.3%) would be excluded based on median QTc. Demographic characteristics were not otherwise associated with the likelihood of meeting any assessed clinical trial exclusion or AHA/ACC thresholds.

**TABLE 5:** Clinical trial corrected QT (QTc) exclusion criteria and numbers of patients in this study who would potentially be excluded from clinical trials.

Exclusion criterion	Number of patients meeting exclusion criterion
**AHA/ACC criteria**	
Median QTc > 450 (men)/460 (women) ms	50 (18.5%)
Median QTc > 470 (men)/480 (women) ms	17 (6.3%)
Median QTc > 500 ms (all patients)	4 (1.5%)
Maximum QTc > 450 (men)/460 (women) ms	140 (51.7%)
Maximum QTc > 470 (men)/480 (women) ms	75 (27.7%)
Maximum QTc > 500 ms (all patients)	31 (11.4%)
**Exclusion criteria described in ClinicalTrials.gov**	
Median QTc > 450 ms (all patients)	74 (27.3%)
Median QTc > 470 ms (all patients)	23 (8.5%)
Median QTc > 480 ms (all patients)	8 (3.0%)
Median QTc > 500 ms (all patients)	4 (1.5%)
Maximum QTc > 450 ms (all patients)	157 (57.9%)
Maximum QTc > 470 ms (all patients)	88 (32.5%)
Maximum QTc > 480 ms (all patients)	60 (22.1%)
Maximum QTc > 500 ms (all patients)	31 (11.4%)

AHA/ACC scientific statements identify 450 ms (men)/460 ms (women) and 470 ms (men)/480 ms (women) as the 90th and 99th percentiles of the normal QTc intervals, respectively. QTc > 500 ms for all patients was identified as a relevant QTc threshold by both the AHA/ACC and from our survey of ClinicalTrials.gov.

Results from our analyses associating prescriptions for QT-prolonging drugs with the odds of meeting clinical trial exclusionary QTc thresholds are shown in Table 6. Prescriptions for QT-prolonging drugs within 30 days of the index date were associated with increases in the percentage of patients having a maximum QTc that exceeded the > 450 ms threshold (all sexes pooled) identified *via* ClinicalTrials.gov (*p* < 0.001) and the > 450/460 ms thresholds in men and women, respectively, from AHA/ACC scientific statements (*p* = 0.001). Additionally, each QT-prolonging medication prescribed within 30 days increased the odds that a patient’s maximum QTc would exceed the > 450 ms (odds ratio = 1.40, *p* < 0.001), 450/460 ms (odds ratio = 1.30, *p* < 0.001), 470 ms (odds ratio = 1.28, *p* = 0.002), 470/480 ms (odds ratio = 1.20, *p* = 0.010), and 480 ms thresholds (odds ratio = 1.21, *p* = 0.030; Table 7). These associations were similar at the other assessed time points, and each QT-prolonging medication significantly increased the odds that a patient’s maximum QTc exceeded the 500 ms threshold when prescribed at 90 days (odds ratio = 1.24, *p* = 0.030), 60 days (odds ratio = 1.18, *p* = 0.046), or at any time before the index date (odds ratio = 1.13, *p* = 0.030). When individual drugs were considered, prescriptions for ondansetron within 30 days were associated with increased odds of a patient’s maximum QTc exceeding all assessed exclusionary thresholds (Table 4). Prescriptions for promethazine within 30 days were associated with increased odds of a patient’s maximum QTc exceeding the 450 ms, 450/460 ms, and > 470 ms thresholds, and, similarly, propofol prescriptions were associated with increased odds of exceeding the 450 ms and 450/460 ms thresholds. The associations of increased odds of exceeding exclusionary QTc thresholds with prescriptions for ondansetron, promethazine, and propofol were also observed at the other assessed time points. Data for all assessed drugs and time points are provided in Data Sheet 1.

**TABLE 6:** Probability of meeting clinical trial corrected QT (QTc) exclusion criteria based on whether patients were prescribed QT-prolonging drugs within 30 days of the index date.

	Patients prescribed no drugs (*n* = 15)	Patients prescribed only non-QT drugs (*n* = 57)	Patients prescribed QT-prolonging drugs (*n* = 199)	*P*-value (*post hoc P*-value for QT vs. non-QT*)
**AHA/ACC criteria**				
Patients with maximum QTc > 450/460 milliseconds (ms)	4 (26.7%)	19 (33.3%)	116 (58.3%)	**< 0.001 (0.003)**
Patients with maximum QTc > 470/480 ms	0 (0%)	12 (21.1%)	63 (31.7%)	**0.007** (0.42)
Patients with maximum QTc > 500 ms	0 (0%)	3 (5.3%)	28 (14.1%)	0.076 (0.31)
**Exclusion criteria from ClinicalTrials.gov**				
Patients with maximum QTc > 450 ms	5 (33.3%)	20 (35.1%)	132 (66.3%)	**< 0.001 (< 0.001)**
Patients with maximum QTc > 470 ms	1 (6.7%)	13 (22.8%)	74 (37.2%)	**0.010** (0.17)
Patients with maximum QTc > 480 ms	0 (0%)	8 (14.0%)	52 (26.1%)	**0.010** (0.23)
Patients with maximum QTc > 500 ms	0 (0%)	3 (5.3%)	28 (14.1%)	0.076 (0.10)

*Post hoc p-values were Bonferroni-corrected (multiplied by 3) to account for multiple comparisons. Fisher’s exact test was used to compare percentages among groups. AHA/ACC scientific statements identify 450 ms (men)/460 ms (women) and 470 ms (men)/480 ms (women) as the 90th and 99th percentiles of the normal QTc intervals, respectively. QTc > 500 ms for all patients was identified as a relevant QTc threshold by both the AHA/ACC and from our survey of ClinicalTrials.gov. Bold values indicates that the *p*-value is significant at the <0.05 threshold.

**TABLE 7:** Correlation between the number of QT-prolonging drugs prescribed and the odds of meeting clinical trial corrected QT (QTc) exclusion criteria at multiple assessed time points.

Logistic regression: Odds ratio, 95% CI (*p*-value)					

AHA/ACC criteria					

	Any time before	90 days	60 days	30 days	10 days
Maximum QTc > 450/460 milliseconds (ms)	1.1, 1.1–1.2 **(0.001)**	1.3, 1.2–1.4 **(< 0.001)**	1.4, 1.2–1.5 **(< 0.001)**	1.3, 1.2–1.5 **(< 0.001)**	1.4, 1.2–1.6 **(< 0.001)**
Maximum QTc > 470/480 ms	1.1, 1.0–1.2 **(0.040)**	1.2, 1.1–1.4 **(0.003)**	1.3, 1.1–1.4 **(0.002)**	1.2, 1.1–1.4 **(0.010)**	1.3, 1.1–1.6 **(0.003)**
Maximum QTc > 500 ms	1.1, 1.0–1.3 **(0.030)**	1.2, 1.0–1.4 **(0.046)**	1.2, 1.1–1.5 **(0.030)**	1.2, 1.0–1.5 (0.060)	1.2, 1.0–1.5 (0.14)

**Exclusion criteria from ClinicalTrials.gov**					

	**Any time before**	**90 days**	**60 days**	**30 days**	**10 days**

Maximum QTc > 450 ms	1.1, 1.1–1.2 **(0.001)**	1.4, 1.2–1.5 **(< 0.001)**	1.4, 1.3–1.6 **(< 0.001)**	1.4, 1.2–1.6 **(< 0.001)**	1.4, 1.2–1.7 **(< 0.001)**
Maximum QTc > 470 ms	1.1, 1.1–1.2 **(0.002)**	1.3, 1.2–1.5 **(< 0.001)**	1.4, 1.2–1.5 **(< 0.001)**	1.3, 1.1–1.5 **(0.002)**	1.3, 1.1–1.6 **(0.002)**
Maximum QTc > 480 ms	1.1, 1.1–1.2 **(0.007)**	1.2, 1.1–1.4 **(0.004)**	1.3, 1.1–1.4 **(0.004)**	1.2, 1.1–1.4 **(0.030)**	1.3, 1.1–1.5 **(0.020)**
Maximum QTc > 500 ms	1.1, 1.0–1.3 **(0.030)**	1.2, 1.0–1.4 **(0.046)**	1.2, 1.1–1.5 **(0.030)**	1.2, 1.0–1.5 (0.060)	1.2, 1.0–1.5 (0.14)

AHA/ACC scientific statements identify 450 ms (men)/460 ms (women) and 470 ms (men)/480 ms (women) as the 90th and 99th percentiles of the normal QTc intervals, respectively. QTc > 500 ms for all patients was identified as a relevant QTc threshold by both the AHA/ACC and from our survey of ClinicalTrials.gov. Bold values indicates that the *p*-value is significant at the <0.05 threshold.

## Discussion

In this investigation, we demonstrate the potential for the administration of QT-prolonging drugs to impact clinical trial eligibility in a cohort of adults with advanced cancer. Our findings indicate that advanced cancer patients are commonly prescribed QT-prolonging drugs, as evidenced by 99.6% of our cohort having ≥ 1 prescription for a QT-prolonging drug since first cancer diagnosis. We also found that prescriptions for QT-prolonging drugs were robustly associated with prolonged QTc intervals across the many time points assessed in our analyses and in our paired analysis that compared QTc intervals in the same patients when co-prescribed and not co-prescribed QT-prolonging drugs. When considering exclusionary QTc thresholds from ongoing and recently completed clinical trials for cancer therapeutics, we found that (1) over half of our cohort (57.9%) had maximum QTc values that would exclude them from trials with the most stringent QTc thresholds (> 450 ms) and (2) the number of QT-prolonging drugs prescribed increased the odds of meeting exclusionary QTc thresholds by 9–40%. In addition, our analyses identify (1) specific demographic characteristics, including female sex, that were associated with increased prescriptions for QT-prolonging drugs and with greater odds of meeting exclusionary QTc thresholds and (2) specific drugs, including ondansetron, promethazine, and propofol, that were associated with > 10 ms increases in QTc and with increased risk of meeting exclusionary QTc thresholds.

Although we are not aware of previous investigations that have directly assessed the potential for the administration of QT-prolonging drugs to affect cancer trial eligibility, results from past studies do support our findings that QT-prolonging drugs can affect trial eligibility. Past studies have consistently found that prescriptions for QT-prolonging drugs are common in cancer patients and that numerous cancer therapeutics prolong QTc. With regard to the prevalence of prescriptions for QT-prolonging drugs, retrospective studies have found that 17.1% (15), 28.4% (14), and 92.6% (12) of cancer patients were prescribed ≥ 1 QT-prolonging drug as determined by CredibleMeds^®^. The variability in these results likely stems from the type of cancer populations that were studied and the duration of follow-up. The prevalence of prescriptions for ≥ 1 QT-prolonging drug in our cohort (99.6%) is higher than those found in past investigations, and this is likely due to the fact that we studied patients since their date of first cancer diagnosis (median duration of follow-up: 3.0 years), which was longer than study periods from past investigations that ranged from 1 week to 1 year (12, 14, 15).

The most commonly prescribed QT-prolonging drugs in our study were also similar to those from past studies and included antiemetics, antimicrobials, antidepressants, and analgesics (12–15). Past investigations have also demonstrated that cancer therapeutics, including capecitabine, arsenic trioxide, combination epirubicin/cyclophosphamide, vorinostat, and numerous TKIs, are associated with prolonged QTc in greater than 10% of patients, based on the Common Terminology Criteria for Adverse Events thresholds (QTc > 450 ms or increase in QTc > 60 ms from baseline) (16–19). Abu Rmilah, et al. found that 28.8% of patients with mixed cancers treated with TKIs had QTc prolongation, with life-threatening QTc prolongation, including the development of ventricular arrhythmias, occurring in 5.4% of patients (16). Our study expands on these findings by demonstrating that ondansetron, promethazine, and propofol, which are commonly prescribed to cancer patients, were each associated with QTc prolongation of > 10 ms. While the number of patients treated with each drug wasn’t large enough to allow an adequately powered statistical analysis, the median QTc values were > 450 ms in patients receiving a prescription in the preceding 30 days for a number of other medications in our analyses; these included the TKIs lenvatinib, crizotinib, and sunitinib, the anti-androgen degarelix, and the supportive therapies hydroxychloroquine, flecainide, clarithromycin, nortriptyline, nicardipine, tolterodine, methadone, and dextromethorphan.

Finally, a study by Kim, et al. compared QTc intervals between patients with cancer and healthy stem cell donors, and demonstrated that cancer patients had prolonged QTc values (mean Bazett’s-corrected QT was 427 ms in cancer patients and 413 ms in healthy donors) (13). While our investigation only included cancer patients, the median Bazett’s-corrected QT value of 438 ms in our cohort numerically supports the association found by Kim, et al. In addition, our finding suggests that patients with advanced cancer may have further prolonged QTc values relative to the Kim, et al. cancer cohort, which consisted of general cancer patients, though caution is warranted when comparing QTc values among patient populations from different health systems.

Our study also expands upon past investigations to discover novel insights with important implications for clinical oncology. Based on information listed on ClinicalTrials.gov, we found that the most common QTc thresholds used for cancer clinical trial exclusion were 450, 470, and 480 ms (Figure 1). It is noteworthy, and likely not coincidental, that these thresholds correspond to the 95th and 99th percentile values for QTc that are identified by AHA/ACC scientific statements as portending arrhythmia risk (3, 21). Our findings also demonstrate that demographic variables, including female sex, increased the odds of meeting exclusionary QTc thresholds. Although it is well-established that women have longer baseline QTc intervals than men (3, 21, 25), we found that < 10% of cancer trials considered patient sex when setting exclusionary QTc thresholds, which would result in significantly more women being excluded from the majority of trials. Standardized incorporation of sex into exclusionary QTc thresholds, as is done in the AHA/ACC scientific statements, is likely to prevent undue exclusion of women from cancer trials while still minimizing arrhythmia risk. In addition, the number of QTc prolonging medications was also associated with increased odds of meeting exclusionary QTc thresholds. Though demographics are immutable, clinicians can appreciate the increased risk of QTc prolongation in at-risk demographic subgroups; conversely, concomitant prescriptions for QT-prolonging drugs can be clinically managed to reduce the risk of arrhythmia and clinical trial exclusion. Guidance for the management of QTc prolongation in cancer patients recommends therapeutic substitution of QT-prolonging drugs as a major clinical strategy to mitigate QTc prolongation (5, 8). While therapeutic substitution might not always be possible for cancer therapeutics without sacrificing efficacy, our findings support the feasibility of therapeutic substitution for supportive therapies, since non-QT-prolonging alternatives exist for the majority QT-prolonging drugs commonly prescribed in our cohort. Substitution to non-QT-prolonging drugs may involve administering a different drug class (e.g., ondansetron must be substituted to a drug from a different class, like aprepitant, since all serotonin receptor 5-HT_3_ antagonist antiemetics prolong QT), but, in many cases, non-QT-prolonging alternatives exist within the same drug class (e.g., opioid analgesics, selective serotonin reuptake inhibitors). Thus, our findings suggest that therapeutic substitution to non-QT-prolonging alternative drugs may be a viable strategy to enhance clinical trial eligibility for advanced cancer patients.

We acknowledge the following limitations of our investigation. First, all QTc values in our study were collected from ECGs that were obtained during normal clinical care. Since ECGs are more likely to be obtained when cardiac abnormalities are suspected, our data collection methods may have enriched for QTc values that are prolonged relative to those from otherwise healthy adults with cancer undergoing ECG screening before enrollment into cancer clinical trials. Second, our extracted medication data did not include sufficient information to determine the days’ supply for each prescription or whether medications were prescribed on an “as needed” (so-called “PRN”) basis. To account for these limitations, (1) we performed our analyses associating prescriptions for QT-prolonging drugs with QTc values using multiple time points (e.g., 10, 30, and 60, and 90 days) and (2) within our paired analysis that assessed QTc values in patients while co-prescribed and not co-prescribed QT-prolonging drugs, we used conservative methods to estimate the days’ supply for each prescription. Given our findings that prescriptions for QT-prolonging drugs were consistently associated with prolonged QTc values across our analyzed time points and in our paired analysis, we do not believe that limitations related to our medication data meaningfully impacted our results. Additionally, there are a host of clinical factors that are known to prolong the QT interval (26). While we attempted to account for the effect of serum electrolyte abnormalities and extreme heart rates on the QTc values observed in our cohort, these analyses were limited by the fact that electrolyte and heart rate data were not regularly captured simultaneously with QTc measurements in the EHR. Given that electrolyte abnormalities and extreme heart rates were common in our cohort, these factors likely influenced our observed QTc values; however, since we excluded “conditional” QT-prolonging drugs from our analyses (which affect QTc *via* alteration of these clinical factors), we believe our analyses demonstrate the effect of QT-prolonging drugs on cancer trial eligibility independent of these clinical factors.

This investigation demonstrates the potential for the administration of QT-prolonging drugs to limit trial eligibility, based on exclusionary QTc thresholds from current or recently completed cancer clinical trials. In addition, our work identifies specific demographic characteristics and medications that are associated with reduced trial eligibility. Importantly, our findings suggest that therapeutic substitution to non-QT-prolonging alternative drugs may be a potentially viable clinical strategy to enhance trial eligibility. However, prospective studies are needed to validate our findings and to determine the clinical validity of therapeutic substitution.

## Data availability statement

The raw data supporting the conclusions of this article will be made available by the authors, without undue reservation.

## Ethics statement

The studies involving human participants were reviewed and approved by Indiana University Institutional Review Board. The patients/participants provided their written informed consent to participate in this study.

## Author contributions

ER and TS analyzed the data and performed the statistical analyses. ER, TS, RL, and TCS wrote the manuscript. All authors contributed to the conception, design of the study, manuscript revision, read, and approved the submitted version.

## Footnotes

1
www.ClinicalTrials.gov

2
www.crediblemeds.org
