# Supplementary material for: Association of QT interval-prolonging drugs with clinical trial eligibility in patients with advanced cancer
Source: Front Cardiovasc Med. 2022 Dec 15;9:894623. doi: 10.3389/fcvm.2022.894623 (PMC9798408; doi:10.3389/fcvm.2022.894623)
Supplement: Supplementary file 2 [file Data_Sheet_2.docx]

## Supplemental figures and tables


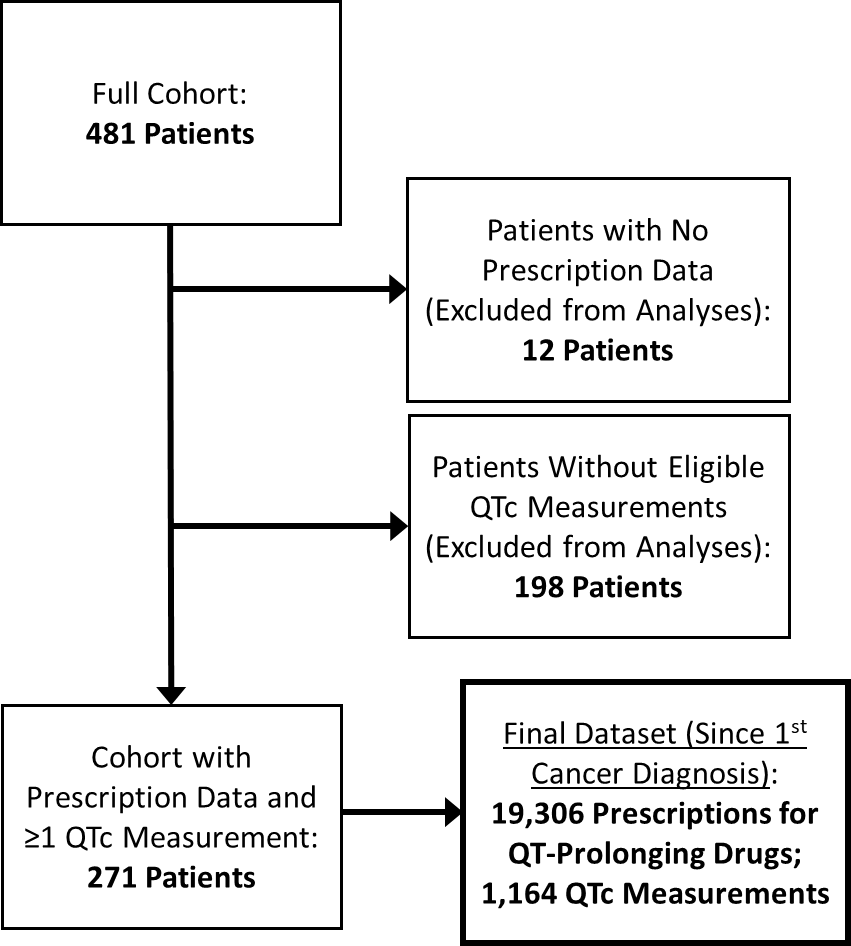
Supplementary Figure 1. Study consort diagram, showing unique numbers of patients, prescriptions for QT-prolonging drugs, and corrected QT (QTc) measurements included in the study.

**Supplementary Figure 2. Change in median corrected QT (QTc) measurements for individual patients (n=160) when prescribed QT-prolonging drugs relative to when not prescribed QT-prolonging drugs. Positive values indicate that median QTcs were prolonged when prescribed QT-prolonging drugs relative to when not. This histogram supplements the data shown in Figure 3 by providing individual patient-level data.**

**Supplementary Table 1. List of International Classification of Diseases and Current Procedural Terminology codes that were queried in the Indiana Health Information Exchange to identify ventricular arrhythmia-related diagnoses or interventions.**

| **Type** | **Code Number** | **Code Description** |
| --- | --- | --- |
| Current Procedural Terminology | 33207 | Insertion of new or replacement of permanent pacemaker with transvenous electrode(s); ventricular |
| Current Procedural Terminology | 33208 | Insertion of new or replacement of permanent pacemaker with transvenous electrode(s); atrial and ventricular |
| International Classification of Diseases | 427.1; I47.2 | Ventricular tachycardia |
| International Classification of Diseases | 427.41; I49.01 | Ventricular fibrillation |
| International Classification of Diseases | 427.5; I46.9 | Cardiac arrest, cause unspecified |
| International Classification of Diseases | I46.8 | Cardiac arrest due to other underlying condition |
| International Classification of Diseases | I49.02 | Ventricular flutter |
| International Classification of Diseases | I49.3 | Ventricular premature depolarization |
| International Classification of Diseases | Z95. 0 | Presence of cardiac pacemaker |
| International Classification of Diseases | Z95.810 | Presence of automatic (implantable) cardiac defibrillator |

**Supplementary Table 2. Days supply assumed for prescriptions dispensed from a pharmacy (used to determine overlap with corrected QT [QTc] measurements for paired analysis).**

| **Drug** | **Assumed Days Supply** |
| --- | --- |
| Alfuzosin | 30 |
| Amiodarone | 7 |
| Azithromycin | 7 |
| Cabozantinib | 30 |
| Capecitabine | 14 |
| Chlorpromazine | 1 |
| Cilostazol | 30 |
| Ciprofloxacin | 7 |
| Citalopram | 30 |
| Crizotinib | 30 |
| Dabrafenib | 30 |
| Encorafenib | 30 |
| Erythromycin | 7 |
| Escitalopram | 30 |
| Flecainide | 30 |
| Fluconazole | 7 |
| Granisetron | 2 |
| Haloperidol | 1 |
| Hydrocodone (extended release) | 7 |
| Hydroxychloroquine | 30 |
| Imipramine | 30 |
| Lenvatinib | 30 |
| Leuprolide | 30 |
| Levofloxacin | 7 |
| Methadone | 7 |
| Mirtazapine | 30 |
| Moxifloxacin | 7 |
| Nortriptyline | 30 |
| Ondansetron | 2 |
| Palonosetron | 1 |
| Pazopanib | 30 |
| Promethazine | 1 |
| Sunitinib | 28 |
| Tacrolimus | 30 |
| Tizanidine | 1 |
| Tolterodine | 30 |
| Tramadol | 7 |
| Venlafaxine | 30 |

**Supplementary Table 3**. **Corrected QT (QTc) measures and probability of meeting clinical trial QTc exclusion based on whether patients were prescribed QT-prolonging drugs within multiple times points before the index date.**

|  |  | Patients Prescribed No Drugs | | Patients Prescribed *Only* Non-QT Drugs | | Patients Prescribed QT-Prolonging Drugs | | | *p* value (post-hoc *p* value for QT vs. Non-QT*) | |  | |
| --- | --- | --- | --- | --- | --- | --- | --- | --- | --- | --- | --- | --- |
| Mean QTc | 90 Days | 437 ± 22, n=12 | | 427 ± 24, n=35 | | 441 ± 29, n=224 | | | **0.003 (0.005)** | |  | |
|  | 60 Days | 438 ± 21, n=15 | | 427 ± 24, n=41 | | 441 ± 27, n=215 | | | **0.002 (0.002)** | |  | |
|  | 30 Days | 438 ± 21, n=15 | | 429 ± 24, n=57 | | 443 ± 28, n=199 | | | **<0.001 (<0.001)** | |  | |
|  | 10 Days | 434 ± 21, n=20 | | 431 ± 26, n=76 | | 443 ± 28, n=175 | | | **0.001 (0.003)** | |  | |
|  | Any Time | 430 ± 28, n=10 | | 431 ± 27, n=13 | | 441 ± 31, n=248 | | | **0.026** (0.056) | |  | |
| Median QTc | 90 Days | 434 ± 23 | | 427 ± 17 | | 441 ± 32 | | | **0.003 (0.004)** | |  | |
|  | 60 Days | 433 ± 22 | | 427 ± 23 | | 441 ± 32 | | | **0.002 (0.002)** | |  | |
|  | 30 Days | 433 ± 22 | | 427 ± 23 | | 442 ± 31 | | | **<0.001 (<0.001)** | |  | |
|  | 10 Days | 432 ± 21 | | 431 ± 29 | | 443 ± 30 | | | **0.001 (0.003)** | |  | |
|  | Any Time | 428 ± 31 | | 431 ± 24 | | 440 ± 31 | | | **0.010 (0.017)** | |  | |
| Maximum QTc | 90 Days | 440 ± 24 | | 435 ± 48 | | 459 ± 39 | | | **<0.001 (0.002)** | |  | |
|  | 60 Days | 441 ± 30 | | 437 ± 48 | | 459 ± 39 | | | **<0.001 (0.002)** | |  | |
|  | 30 Days | 441 ± 30 | | 437 ± 48 | | 460 ± 37 | | | **<0.001 (<0.001)** | |  | |
|  | 10 Days | 440 ± 31 | | 446 ± 44 | | 461 ± 38 | | | **<0.001 (<0.001)** | |  | |
|  | Any Time | 434 ± 26 | | 431 ± 51 | | 457 ± 39 | | | **0.003** (0.075) | |  | |
| Minimum QTc | 90 Days | 422 ± 22 | | 417 ± 27 | | 421 ± 33 | | | 0.19 (0.34) | |  | |
|  | 60 Days | 424 ± 20 | | 416 ± 29 | | 420 ± 34 | | | 0.072 (0.20) | |  | |
|  | 30 Days | 424 ± 20 | | 416 ± 25 | | 421 ± 36 | | | 0.084 (0.24) | |  | |
|  | 10 Days | 423 ± 24 | | 420 ± 27 | | 420 ± 34 | | | 0.56 (1.00) | |  | |
|  | Any Time | 422 ± 23 | | 419 ± 29 | | 420 ± 31 | | | 0.52 (1.00) | |  | |
| Difference Between Maximum & Minimum QTc | 90 Days | 0 ± 20 | | 8 ± 38 | | 34 ± 50 | | | **<0.001 (0.032)** | |  | |
|  | 60 Days | 0 ± 24 | | 19 ± 37 | | 35 ± 51 | | | **<0.001 (0.043)** | |  | |
|  | 30 Days | 0 ± 24 | | 17 ± 37 | | 37 ± 53 | | | **<0.001 (0.005)** | |  | |
|  | 10 Days | 0 ± 20 | | 18 ± 48 | | 39 ± 48 | | | **<0.001 (0.003)** | |  | |
|  | Any Time | 0 ± 15 | | 0 ± 31 | | 34 ± 51 | | | **0.001** (0.052) | |  | |
| Patients with Maximum QTc >450 | 90 Days | | 3 (25%) | | 12 (34.3%) | | 142 (63.4%) | **<0.001 (0.004)** | |  | |  |
|  | 60 Days | | 5 (33.3%) | | 14 (34.1%) | | 138 (64.2%) | **<0.001 (0.001)** | |  | |  |
|  | 30 Days | | 5 (33.3%) | | 20 (35.1%) | | 132 (66.3%) | **<0.001 (<0.001)** | |  | |  |
|  | 10 Days | | 6 (30%) | | 34 (44.7%) | | 117 (66.9%) | **<0.001 (0.004)** | |  | |  |
|  | Any Time | | 2 (20%) | | 5 (38.5%) | | 150 (60.5%) | **0.013** (0.45) | |  | |  |
| Patients with Maximum QTc >450/460 | 90 Days | | 2 (16.7%) | | 12 (34.3%) | | 125 (55.8%) | **0.002** (0.055) | |  | |  |
|  | 60 Days | | 4 (26.7%) | | 14 (34.1%) | | 121 (56.3%) | **0.004 (0.031)** | |  | |  |
|  | 30 Days | | 4 (26.7%) | | 19 (33.3%) | | 116 (58.3%) | **<0.001 (0.003)** | |  | |  |
|  | 10 Days | | 5 (25.0%) | | 29 (38.2%) | | 105 (60.0%) | **<0.001 (0.004)** | |  | |  |
|  | Any Time | | 2 (20.0%) | | 4 (30.8%) | | 133 (53.6%) | **0.035** (0.15) | |  | |  |
| Patients with Maximum QTc >470 | 90 Days | | 0 (0%) | | 8 (22.9%) | | 80 (35.7%) | **0.009** (0.54) | |  | |  |
|  | 60 Days | | 1 (6.7%) | | 10 (24.4%) | | 77 (35.8%) | **0.027** (0.62) | |  | |  |
|  | 30 Days | | 1 (6.7%) | | 13 (22.8%) | | 74 (37.2%) | **0.01** (0.17) | |  | |  |
|  | 10 Days | | 2 (10.0%) | | 17 (22.4%) | | 69 (39.4%) | **0.002 (0.028)** | |  | |  |
|  | Any Time | | 0 (0%) | | 3 (23.1%) | | 85 (34.3%) | **0.049** (1.00) | |  | |  |
| Patients with Maximum QTc >470/480 | 90 Days | | 0 (0%) | | 7 (20.0%) | | 68 (30.4%) | **0.024** (0.71) | |  | |  |
|  | 60 Days | | 0 (0%) | | 10 (24.4%) | | 65 (30.2%) | **0.022** (1.00) | |  | |  |
|  | 30 Days | | 0 (0%) | | 12 (21.1%) | | 63 (31.7%) | **0.007** (0.42) | |  | |  |
|  | 10 Days | | 1 (5%) | | 15 (19.7%) | | 59 (33.7%) | **0.003** (0.10) | |  | |  |
|  | Any Time | | 0 (0%) | | 3 (23.1%) | | 72 (29.0%) | 0.117 (1.00) | |  | |  |
| Patients with Maximum QTc >480 | 90 Days | | 0 (0%) | | 4 (11.4%) | | 56 (25.0%) | **0.028** (0.26) | |  | |  |
|  | 60 Days | | 0 (0%) | | 6 (14.6%) | | 54 (25.1%) | **0.026** (0.49) | |  | |  |
|  | 30 Days | | 0 (0%) | | 8 (14.0%) | | 52 (26.1%) | **0.01** (0.23) | |  | |  |
|  | 10 Days | | 1 (5%) | | 10 (13.2%) | | 49 (28.0%) | **0.005** (**0.043**) | |  | |  |
|  | Any Time | | 0 (0%) | | 2 (15.4%) | | 58 (23.4%) | 0.198 (1.00) | |  | |  |
| Patients with Maximum QTc >500 | 90 Days | | 0 (0%) | | 2 (5.7%) | | 29 (12.9%) | 0.301 (0.83) | |  | |  |
|  | 60 Days | | 0 (0%) | | 3 (7.3%) | | 28 (13.0%) | 0.253 (1.00) | |  | |  |
|  | 30 Days | | 0 (0%) | | 3 (5.3%) | | 28 (14.1%) | 0.076 (0.31) | |  | |  |
|  | 10 Days | | 1 (5%) | | 4 (7.0%) | | 26 (14.9%) | 0.056 (0.10) | |  | |  |
|  | Any Time | | 0 (0%) | | 1 (7.7%) | | 30 (12.1%) | 0.76 (1.00) | |  | |  |

*Post-hoc *p* values were Bonferroni-corrected (multiplied by 3) to account for multiple comparisons.

Notes: Number of patients in each category at each time point were the same as shown in the “Mean QTc” section. All QTc values are in milliseconds. Data are presented as median ± interquartile range and percentages. Kruskal-Wallis test was used to compare continuous data, and Fisher’s exact test with post hoc comparisons for paired groups was used to compare percentages.
